# Selection for longer lived sperm within ejaculate reduces reproductive ageing in offspring

**DOI:** 10.1002/evl3.101

**Published:** 2019-02-14

**Authors:** Ghazal Alavioon, Andrea Cabrera Garcia, Magali LeChatelier, Alexei A. Maklakov, Simone Immler

**Affiliations:** ^1^ Department of Ecology and Genetics, Evolutionary Biology Centre Uppsala University Norbyvägen 18D 75 236 Uppsala Sweden; ^2^ School of Biological Sciences University of East Anglia Norwich Research Park Norwich NR4 7TJ United Kingdom

**Keywords:** Ageing, reproductive success, sperm selection, sperm competition, senescence

## Abstract

Males produce numerous sperm in a single ejaculate that greatly outnumber their potential egg targets. Recent studies found that phenotypic and genotypic variation among sperm in a single ejaculate of a male affects the fitness and performance of the resulting offspring. Specifically, within‐ejaculate sperm selection for sperm longevity increased the performance of the resulting offspring in several key life‐history traits in early life. Because increased early‐life reproductive performance often correlates with rapid ageing, it is possible that within‐ejaculate sperm selection increases early‐life fitness at the cost of accelerated senescence. Alternatively, within‐ejaculate sperm selection could improve offspring quality throughout the life cycle, including reduced age‐specific deterioration. We tested the two alternative hypotheses in an experimental setup using zebrafish *Danio rerio*. We found that within‐ejaculate sperm selection for sperm longevity reduced age‐specific deterioration of fecundity and offspring survival but had no effect on fertilization success in males. Remarkably, we found an opposing effect of within‐ejaculate sperm selection on female fecundity, where selection for sperm longevity resulted in increased early‐life performance followed by a slow decline, while females sired by unselected sperm started low but increased their fecundity with age. Intriguingly, within‐ejaculate sperm selection also reduced the age‐specific decline in fertilization success in females, suggesting that selection for sperm longevity improves at least some aspects of female reproductive ageing. These results demonstrate that within‐ejaculate variation in sperm phenotype contributes to individual variation in animal life histories in the two sexes and may have important implications for assisted fertilization programs in livestock and humans.

**Impact Summary**

One male produces thousands to millions of sperm in a single ejaculate but only very few end up fertilizing an egg. The sperm within an ejaculate vary not only in their shape and performance, but also in the genetic material that each of them carries. The variation among sperm within an ejaculate has long been thought to be of little consequence for the resulting offspring. However, here we show that when we select for the longer lived sperm within the ejaculate of male zebrafish, the resulting offspring is much fitter than their full siblings sired by the shorter lived sperm of the same male. More specifically, offspring sired by longer lived sperm produce more and healthier offspring throughout their life and age at a slower rate. This is a surprising result, which suggests that it is important to understand how sperm selection may contribute to the fitness of the next generations. Our findings not only have important implications for evolutionary biology but potentially beyond into areas that use assisted fertilization technologies.

Sexual reproduction in eukaryotes requires the alteration between a diploid and a haploid phase (Mable and Otto 1998). A large majority of animals spend most of their life as diploid organisms, which alternates with a very short haploid gametic phase. Nevertheless, even a short phase may offer a window for selection to act upon, particularly in male gametes that vary substantially in phenotypes (Holt and Van Look 2004) and genotypes (Wang et al. 2012) and are often exposed to various environmental challenges (Birkhead et al. 1993; Keller and Reeve 1995). However, a long‐standing and common belief holds that selection on mature sperm is of little consequence for the following generation because gene expression is thought to be minimal at the post‐meiotic stages (Eddy 2002). Nevertheless, empirical evidence for gene expression in haploid spermatids is increasing (Fujimoto et al. 1984; Barreau et al. 2008) and the scope for haploid gene expression and hence selection may be more important than assumed so far (Joseph and Kirkpatrick 2004; Immler 2008; Immler and Otto 2018). Even though the haploid phase is rather short in predominantly diploid organisms, selection occurring during the haploid gametic phase may have far reaching consequences for basic evolutionary processes including the rate of adaptation (Orr and Otto 1994), the genetic load (Charlesworth and Charlesworth 1987; Otto et al. 2015), and genetic variation more generally (Immler et al. 2012; Immler and Otto 2018). However, empirical evidence for these effects is still scarce. Selection on longer lived sperm within the ejaculates of a marine ascidian *Styela plicata* resulted in an increase in hatching success and survival (Crean et al. 2012). A similar study in the Atlantic salmon *Salmo salar* showed effects of sperm longevity on time until hatching (Immler et al. 2014). More recently, a study in the zebrafish *Danio rerio* provided strong evidence for the impact of selection at the haploid sperm level on offspring survival and reproductive success and linked sperm phenotype to sperm genotype (Alavioon et al. 2017). These findings suggest that selection on sperm is likely to have a general impact on offspring fitness and will affect an organism throughout life. In this study, we set out to further test how selection on sperm longevity affects life history traits in male and female offspring.

Previous studies suggest that within‐ejaculate sperm selection on sperm longevity has positive effects on a number of important life‐history traits in the resulting progeny, such as early‐life survival, embryo viability, cell apoptosis, and reproductive fitness of male offspring (Alavioon et al. 2017). Specifically, adult males sired by longer lived sperm produced more and faster swimming sperm, which resulted in a higher fertilization success and more offspring early in life when spawning with control females compared to their male siblings sired by unselected sperm. Because the “disposable soma” theory of ageing (Kirkwood et al. 1979; Kirkwood and Austad 2000) states that ageing results from the resource allocation trade‐offs between investment in reproduction and investment in somatic maintenance, and because increased investment in early‐life fitness can come at the cost of late‐life fitness (Lemaître et al. 2015), it is reasonable to predict that within‐ejaculate sperm selection can contribute to accelerated ageing in both sexes, but especially in males. Sexual selection theory suggests that because male reproductive success is more variable than female reproductive success (Andersson 1994; Bateman 1948), males stand to gain more than females from increased investment in early‐life reproduction to the detriment of their long‐life reproduction and survival (Bonduriansky et al. 2008; Maklakov and Lummaa 2013). Indeed, several studies suggested that increased investment in sperm quality trades off with other costly traits, such as immune response (Simmons 2012), which can have negative implications for survival. For example, one recent study suggested that increased sperm competition led to the evolution of male semelparity in marsupials (Fisher et al. 2013). Such a “live fast, die young” reproductive strategy can result in rapid reproductive ageing in high‐quality males (Hunt et al. 2004; Hooper et al. 2018).

However, the payoffs from strategic investment in early‐life reproduction for males depend on the ecology and the mating system of the species and are far from being universal (Hooper et al. 2018). Therefore, it is also possible that within‐ejaculate sperm selection selects for higher quality offspring of both sexes that will exhibit better reproductive performance throughout their life cycle and will thus not only enjoy increased fitness but also decelerated reproductive ageing. Here, we studied the effect of within‐ejaculate sperm selection on sperm longevity on age‐specific life histories of male and female zebrafish *Danio rerio*. We were primarily interested in the two following questions: (i) Does within‐ejaculate sperm selection accelerate or decelerate reproductive ageing? (ii) Does within‐ejaculate sperm selection affect male and female offspring differently? In order to address these questions, we investigated the effect of within‐ejaculate sperm selection on sperm longevity on age‐specific fertilization success, embryo survival, and fecundity in the two sexes.

## Materials and Methods

### ANIMAL MODEL

For all experiments, we used zebrafish *Danio rerio* from the outbred wild‐type AB strain originally obtained from ZIRC (Zebrafish International Resource Center, University of Oregon, Eugene, USA) and maintained at the SciLifeLab zebrafish platform at Uppsala University (http://www.scilifelab.se/facilities/zebrafish/) for up to two generations following a strict outbreeding regime. The fish were kept in 3‐L tanks in a recirculating rack system (Aquatic Habitats, Beverly, MA, USA, Z‐Hab System) at 26.4 ± 1.4°C and a 12:12 diurnal light cycle. They were fed two to three times a day with a mixture of dry food (Aquatic Habitats, Zeigler adult zebrafish diet) and live artemia (ZM Brineshrimp Cysts).

### IN VITRO FERTILIZATIONS

All the fish used for in vitro fertilizations (IVF) and outcrosses were kept in 3‐L tanks at densities of 15–20 fish in mixed sex groups. One day before the experiment, the males were separated into unisexual groups of three and kept overnight. The experimental females were kept in breeding tanks with a company male using a separator (smell and visual contact). Each tank containing experimental females was covered by a black cloth until next morning (to avoid light‐induced oviposition). Males and females were not fed for 20 h before the experiment to avoid fecal contamination of sperm and egg samples.

Females and males were anesthetized using 1.0–3.0 mg/L metomidate hydrochloride (Aquacalm^TM^) or in 0.16 g/L Tricaine methanesulfonate (MS222; Sigma–Aldrich). Males were placed on a soft and wet sponge and squeezed gently in cranio‐caudal direction to collect the ejaculate under a dissecting microscope (Nikon SMZ800). From each male, 0.7–0.8 μL of ejaculate were collected and transferred into a 0.2 mL Eppendorf tube containing 80 μL of Hank's buffer (HBSS) and kept on ice for 5–10 min until IVF. Females were placed on 15 cm Petri dishes and gently stripped to obtain eggs. Clutches used for IVFs contained 20–300 high quality eggs and they were used within one minute after stripping.

### SPERM SELECTION AND IVFS

To create the first generation (F1), we used a split clutch design to perform IVFs. Sperm samples were very gently mixed and each ejaculate and egg clutch were divided into two parts. In each part, the sperm were under one of two treatments. In the long‐activation time (LAT) treatment, 25 μL of ejaculate‐Hank's mix of a male were activated with 400 μL of water and added to one half of each clutch 25 s after activation to obtain a 50% decline in the amount of motile sperm (see Alavioon et al. 2017 for supporting data). In the short‐activation treatment (SAT), 10 μL of ejaculate‐Hank's mix from the same male were mixed with extra 15 μL of HBSS (to compensate for osmolarity in HBSS to water ratio), activated with 400 μL water and added to the other sub‐clutch immediately after activation. Activated sperm in both treatments were added simultaneously to both sub‐clutches to avoid any egg effect. In the LAT treatment, we selected against sperm with longevity less than 25 s while in the SAT treatment, eggs could be fertilized by any sperm capable of fertilization (for experimental details see Supporting Information). Applying a split clutch design enabled us to compare the effect of sperm selection on reproductive ageing between full siblings sired by sperm of different phenotype and presumably genotype. Eggs and sperm were mixed gently using a brush and eggs were transferred onto a 15 cm Petri dish containing a Methylene blue solution (Sigma Aldrich, anti fungus) after 1 min and 30 s. All IVFs were performed on a warming plate (Minitube HT50) at 28.5°C and all Petri dishes were transferred to an incubator set to 28.5°C.

### REARING F1 OFFSPRING

The embryos resulting from IVFs were checked 2–4 h post fertilization (hpf). Unfertilized and bad quality eggs were removed and about 70 embryos were transferred to a 9 cm Petri dish containing Methylene blue solution (antifungus treatment). On day 5–6 post fertilization, 70 larvae from each family were transferred into a 3‐L tank in the zebrafish system at the facility and reared until adulthood. All fish were maintained until a maximum age of 24 months. Fish that survived until that age were then humanely killed with an overdose of MS222. Captive zebrafish have their reproductive peak between 6 and 18 months of age. For ethical and health reasons, fish can only be maintained until this age to reduce the risk of disease in the facility accumulating at an increased rate in fish older than 24 months.

### NATURAL SPAWNING

Starting at the age of 12 months, experimental males and females from F1 were setup for natural spawnings with wild‐type fish to assess reproductive success. All the fish used for outcrosses with SAT and LAT F1 fish at each age point were wild‐type AB fish bred in the facility following a careful breeding regime to maintain outcrossing. They were maintained in 10‐L tanks at densities of 30–40 fish in mixed sex groups at 1:1 sex ratio. The SAT and LAT fish were kept in 10‐L tanks at densities of 30–40 fish, both in mixed sex groups. One day before natural spawning, males and females were randomly chosen from both, selection and wild‐type lines. One male from either of the selection lines and one female from wild‐type lines or vice versa were kept in a breeding tank with a separator in between. The next morning, the separator was taken out at 8:30–8:45 am to let the two fish spawn. Eggs were collected 2–3 h after spawning.

Two to four males and two to four females were randomly selected from 26 LAT and SAT families. The same families were chosen in each selection regime for the comparison of full siblings across treatments and reduce variation between families. Fish were individually tagged by using Visible Implant Elastomer (VIE; BIOWEB) for tracking over time. Each fish was randomly assigned wild‐type AB partners aged 8–12 months for natural spawning as described above. The same procedure was repeated for all experimental fish at age 12, 15, 18, and 24 months, but every time, we used a new partner fish between 8 and 12 months old. In total, we were able to observe 116 fish from SAT and 111 fish from LAT at age 12 months; 105 fish from SAT and 107 fish from LAT at age 15 months; 98 fish from SAT and 99 fish from LAT at age 18 months; 77 fish from SAT and 76 fish from LAT at age 24 months. All tagged fish were individually followed and checked upon on a daily basis and natural or accidental death was recorded.

### OFFSPRING TRAITS MEASUREMENTS

To calculate the fertilization success, eggs were checked within one hour post fertilization. To calculate fertilization success and fecundity, unfertilized and bad quality eggs were counted and removed. Survival rate was checked at 24 h post fertilization. Seventy embryos were transferred to a 15 cm Petri dish containing a Methylene blue solution and moved back to the incubator set to at 28.5°C. All the traits were measured at 12, 15, 18, and 24 months.

### STATISTICAL ANALYSIS

Software R version 3.3.0 was used for all analysis. We performed a survival analysis to establish differences in lifespan between the treatment and the sexes. In order to do so, we performed Cox models using the package *coxme*. For analyses of traits related to reproductive fitness, all binomial traits were analyzed assuming a binomial error distribution, whereas total egg production was analyzed assuming a Poisson error distribution. We used generalized linear mixed effect models for all analyses (*glmer* function in package *lme4*). The significance of the fitted model was assessed using analysis of variance (ANOVA) with type III sums of squares tested with an analysis of deviance on a chi‐square distribution using package *car*.

We defined “family” and individual “ID” as a random factor and “Treatment,” “Age at Last Reproduction (ALR),” and “Age” as fixed factors. We analyzed data for males and females separately, because the traits that were measured represent different biological traits in the two sexes and are not directly comparable. All the interactions in the final models were assessed performing backward selection (removing model terms starting with interactions with highest order term). At each level, the model was compared to the previous model running *Anova* function in package *car*. Age and ALR were scaled in all models and optimizer “bobyqa” was used in all models except total number of eggs (fecundity).

### ETHICS PERMIT

All experiments described here are in accordance with the guidelines and approved by the Swedish Board of Agriculture (Jordbruksverket approval number C3/15).

## Results

**Figure 1:**
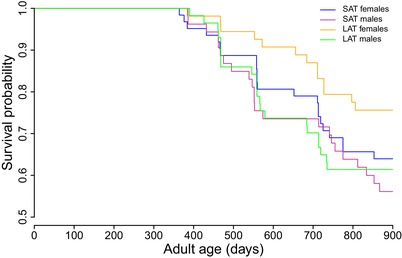
Lifespan data for all fish up to 24 months of age is shown with a clearly reduced lifespan in SAT females compared to LAT females but a less clear difference between SAT and LAT males.

Overall, we found striking effects of our sperm selection treatment on the rate of reproductive ageing in the F1 offspring. In general, SAT offspring exhibited a shorter overall lifespan than the LAT offspring, particularly in females (Treatment: log likelihood value = –411.16, *χ* ^2^_1_ = 11.36, *p* = 0.007; Sex: log likelihood value = –409.18, *χ* ^2^_1_ = 3.95, *p* = 0.046; Fig. 1). While the strength of the effect differed between the sexes, the overall pattern was the same as indicated by a nonsignificant interaction term. In addition, within‐ejaculate sperm selection affected lifetime reproductive success in both sexes, but the effects differed markedly between males and females.

### FERTILIZATION SUCCESS

Fertilization success generally declined with age in both sexes and both treatments. However, sperm selection decelerated female senescence in fertilization success but had no effect on this trait in males.

### FEMALES

**Figure 2:**
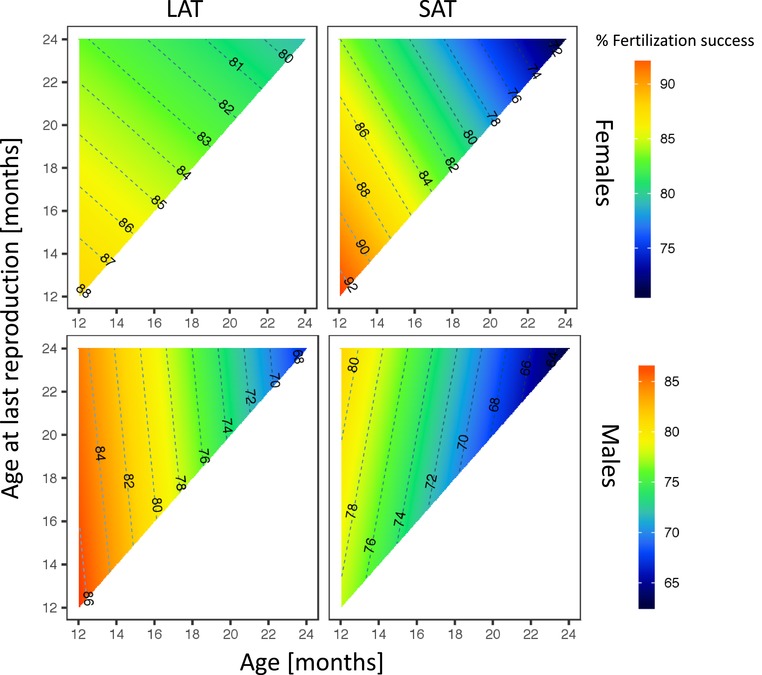
The effect of within‐ejaculate sperm selection on age‐specific fertilization success in males and females in SAT and LAT treatments. Fertilization success declines with age but the pattern differs between the sexes. The decline is smaller in LAT females than SAT females, while fertilization success is higher in LAT males than SAT males.

SAT females that achieved highest fertilization success rates early in life (12 months) lived a shorter life, while LAT females invested moderately into reproduction at early ages but exhibited a longer lifespan (Treatment: *χ* ^2^_1_ = 0.52, *p* = 0.47; Age: *χ* ^2^_1_ = 570.08, *p* < 0.0001; ALR: *χ* ^2^_1_ = 0.01, *p* = 0.92; Treatment × Age: *χ* ^2^_1_ = 62.93, *p* < 0.0001; Treatment × ALR: *χ* ^2^_1_ = 0.15, *p* = 0.70: Age × ALR: *χ* ^2^_1_ = 2.06, *p* = 0.15;Treatment × Age × ALR: *χ* ^2^_1_ = 113.71, *p* < 0.0001; Fig. 2). Female LAT offspring showed relatively little decline in age‐specific fertilization success (∼4%) compared to SAT females (∼14%).

### MALES

In males, fertilization success rate also declined with age and fertilization success rate was higher in LAT males early in life (Treatment: *χ* ^2^_1_ = 2.04, *p* = 0.15; Age: *χ* ^2^_1_ = 556.45, *p* < 0.0001; ALR: *χ* ^2^_1_ = 1.08, *p* = 0.30; Treatment × Age: *χ* ^2^_1_ = 6.80, *p* = 0.009; Treatment × ALR: *χ* ^2^_1_ = 0.25, *p* = 0.62: Age × ALR: *χ* ^2^_1_ = 12.12, *p* = 0.0005; Treatment × Age × ALR: *χ* ^2^_1_ = 19.87, *p* < 0.0001; Fig. 2). Fertilization success dropped by 16–18% in males from both treatments across the monitored lifespan and was on average higher in LAT males than in SAT males. In both treatments, fertilization success rate in males peaked early in life (at 12 months) and then steadily declined until the age of 24 months.

### EMBRYO SURVIVAL

Similarly, embryo survival declined with increasing age in males and females, but the patterns differed between the treatments.

### FEMALES

**Figure 3:**
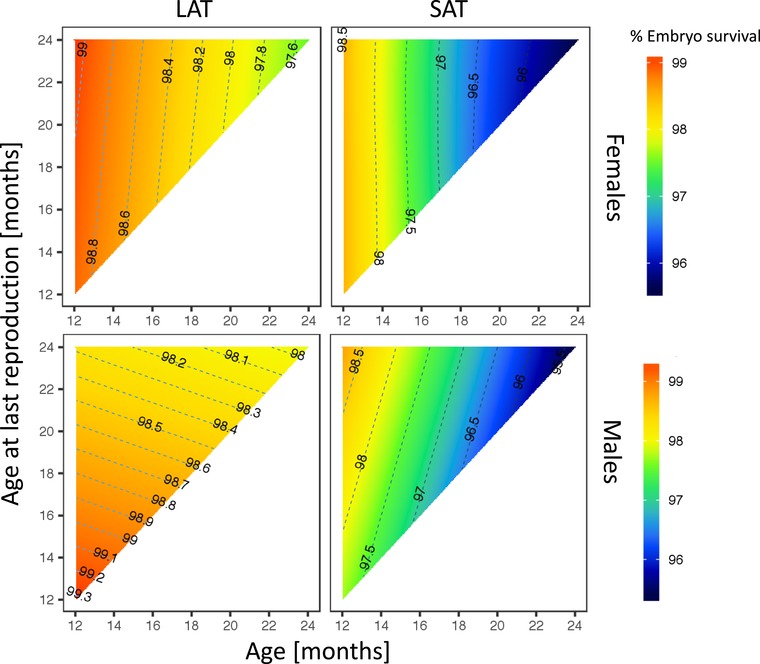
The effect of within‐ejaculate sperm selection on age‐specific embryo survival in males and females in SAT and LAT treatments. Both, LAT males and females had higher embryo survival rate but there was less of a decline with age in LAT offspring than in SAT offspring of both sexes.

In females, higher offspring survival at an early age did not result in reduced lifespan, but this negative association was observed in males. All females exhibited higher rates of embryo survival at early ages with a peak at age 12 months irrespective of overall lifespan. However, embryo survival was generally higher in LAT females than in SAT females, and there was little evidence for a difference in age‐specific rates of decline between LAT (1.3%) and SAT (3%) females (Treatment: *χ* ^2^_1_ = 1.14, *p* = 0.29; Age: *χ* ^2^_1_ = 145.51, *p* < 0.0001; ALR: *χ* ^2^_1_ = 0.17, *p* = 0.68; Treatment × Age: *χ* ^2^_1_ = 4.23, *p* = 0.04; Treatment × ALR: *χ* ^2^_1_ = 0.04, *p* = 0.84: Age × ALR: *χ* ^2^_1_ = 169.05, *p* < 0.0001;Treatment × Age × ALR: *χ* ^2^_1_ = 15.94, *p* < 0.0001; Figure 3).

### MALES

In SAT males, high embryo survival was associated with a longer lifespan, whereas shorter lived SAT males produced less viable offspring. In contrast, high embryo survival in LAT males was coupled with a shorter lifespan. The decline of embryo survival rate with ageing was faster in SAT males (3%) and almost negligible in LAT males (0.7%; Treatment: *χ^2^*_1_ = 2.93, *p* = 0.09; Age: *χ^2^*_1_ = 1.08, *p* = 0.30; ALR: *χ^2^*_1_ = 0.08, *p* = 0.78; Treatment × Age: *χ^2^*_1_ = 6.25, *p* = 0.01; Treatment × ALR: *χ^2^*_1_ = 2.11, *p* = 0.15: Age × ALR: *χ^2^*_1_ = 13.36, *p* = 0.0003; Treatment × Age × ALR: *χ^2^*_1_ = 7.35, *p* < 0.0001; Figure 3).

### FECUNDITY

Finally, the most dramatic difference in age‐specific reproductive performance between the sexes was found in fecundity. Both, male and female LAT offspring had higher fecundity values than their SAT counterparts throughout their reproductive life cycle.

### FEMALES

**Figure 4:**
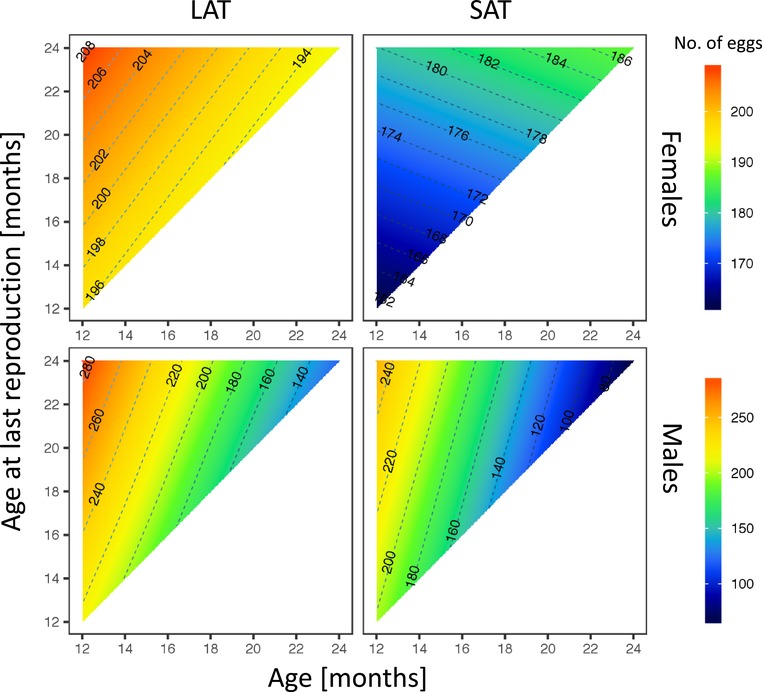
The effect of within‐ejaculate sperm selection on age‐specific fecundity (number of eggs) in males and females in SAT and LAT treatments. LAT offspring had higher fecundity compare to their SAT siblings. SAT females produced higher numbers of eggs at later ages while there was a decline in fecundity in other groups.

While in LAT females the reproductive output declined very slowly across the 12 months period, SAT females slightly increased their reproductive output with age (Treatment: *χ^2^*_1_ = 2.93, *p* = 0.09; Age: *χ^2^*_1_ = 31.22, *p* < 0.0001; Age^2^: *χ^2^*_1_ = 280.73, *p* < 0.0001; ALR: *χ^2^*_1_ = 3.69, *p* = 0.06; Treatment × Age: *χ^2^*_1_ = 7.71, *p* = 0.006; Treatment × Age^2^: *χ^2^*_1_ = 437.12, *p* < 0.0001; Treatment × ALR: *χ^2^*_1_ = 0.02, *p* = 0.88: Age × ALR: *χ^2^*_1_ = 1990.02, *p* < 0.0001; Treatment × Age × ALR: *χ^2^*_1_ = 266.47, *p* < 0.0001; Figure 4).

### MALES

Fecundity in LAT males also declined slower with age (roughly halved their reproductive output from 280 eggs at 12 months to 140 eggs at 24 months laid by mated females) than in SAT males (roughly three times lower reproduction from 240 to 80 eggs at the same time period; Treatment: *χ^2^*_1_ = 1.67, *p* = 0.20; Age: *χ* ^2^_1_ = 5792.52 *p* < 0.0001; Age^2^: *χ^2^*_1_ = 16.95, *p* < 0.0001; ALR: *χ* ^2^_1_ = 16.09, *p* < 0.0001; Treatment × Age: *χ* ^2^_1_ = 247.32, *p* < 0.0001; Treatment × Age^2^: *χ^2^*_1_ = 9.48, *p* = 0.002; Treatment × ALR: *χ* ^2^_1_ = 1.31, *p* = 0.25: Age × ALR: *χ* ^2^_1_ = 689.86, *p* < 0.0001; Treatment × Age × ALR: *χ* ^2^_1_ = 14.47, *p* = 0.0001; Figure 4).

## Discussion

Overall, our results do not support the hypothesis that sperm selection within a single ejaculate results in offspring that show high fitness and rapid ageing. Instead, our findings suggest that within‐ejaculate sperm selection on sperm longevity produces offspring that have higher fitness and longer lifespan during the first 24 months and enjoy lower rates of reproductive ageing (Table 1). The effects on reproductive traits are sex‐specific and vary markedly across the studied traits. Nevertheless, within‐ejaculate sperm selection resulted in male offspring that showed slower decline in fecundity and offspring quality with age, while in females within‐ejaculate sperm selection positively affected egg quality in late life, as evidenced by increased fertilization success in older LAT females compared to SAT females. Lifespan was longer in LAT offspring of both sexes than in SAT offspring with a slightly stronger effect in females but no significantly different pattern.

**Table 1:** Summary of the effect of within‐ejaculate sperm selection on offspring reproductive performance in both sexes. Reproductive lifespan was deducted from our measurements of Age at Last Reproduction (ALR)

Trait	Sex	Treatment	Reproductive peak/reproductive lifespan	Ageing rate
Fertilization success	Female	SAT	Young/short	SAT > LAT
		LAT	Young/short	
	Male	SAT	Young/intermediate	SAT = LAT
		LAT	Young/intermediate	
Embryo survival	Female	SAT	Young/intermediate	SAT > LAT
		LAT	Young/intermediate	
	Male	SAT	Young/intermediate	SAT > LAT
		LAT	Young/short	
Total eggs	Female	SAT	Old/intermediate	LAT > SAT
		LAT	Young/long	
	Male	SAT	Young/long	SAT > LAT
		LAT	Young/long	

The effect of within‐ejaculate sperm selection on male age‐specific reproductive performance was relatively consistent across all three traits. LAT males had better fertilization success, embryo survival, and total fecundity than SAT males at every age. Interestingly, in the high‐quality LAT cohort, short‐lived males showed higher early‐life performance than their long‐lived counterparts in embryo survival, suggesting that short‐lived males adopted a “live fast, die young” reproductive strategy. In contrast, in the SAT cohort, long‐lived males exhibited higher early‐life reproductive performance with respect to embryo survival. These results suggest some form of condition dependence where high‐quality LAT males have different resource allocation strategies than low‐quality SAT males in some traits, underscoring the fact that within‐ejaculate sperm selection shapes age‐specific life histories of the resulting offspring.

The effect of within‐ejaculate sperm selection on female reproductive traits was more variable. While LAT females aged slower than SAT females in terms of fertilization success, the only trait–cohort combination showing improvement with age was fecundity in SAT females. Generally, LAT females produced more eggs than SAT females throughout life. However, in absolute terms, old SAT females still laid fewer eggs than old LAT females; combined with reduced embryo survival of old SAT females, the late‐life fitness of SAT females appears to be quite low. The fact that old SAT females have low fertilization success and low embryo survival suggests that they simply lay many infertile eggs in late life. Because the quality of SAT females’ eggs declined, as suggested by the results showing reduced fertilization success and embryo survival, SAT females strategically allocate relatively more resources to egg production toward the end of their life, which compensates for the decline in their general reproductive state and reduced oocyte quality.

Interestingly, females that reproduced only once or twice at the ages of 12 and 14 months showed highest values for fertilization success, suggesting that these low‐quality individuals may have strategically allocated a substantial amount of resources into early‐life reproduction. This effect is similar to the effect within‐ejaculate selection on embryo survival in LAT males (see above). These findings suggest a potential trade‐off between investments in early‐life versus late‐life performance at least in some reproductive traits in both sexes. Nevertheless, these putative trade‐offs within LAT and SAT cohorts were not sufficiently strong to mask the much more pronounced differences between the two treatments. For example, despite the fact that long‐lived LAT males had lower early life fertilization success than short‐lived LAT males, the latter still aged slower that long‐lived SAT males.

While increased sperm competition between different males can result in the evolution of trade‐offs between sperm quantity and quality versus other traits, such as immune response (Simmons 2012), we currently have no evidence for any trade‐off between selection for sperm longevity and offspring fitness with any other trait. In a previous study, we looked for a trade‐off between sperm swimming speed and sperm longevity, but did not find any (Alavioon et al. 2017). Whether a trade‐off may be found in any other aspect is currently unclear. It may well be that traits other than swimming speed are key in determining fertilization success in the zebrafish. Future investigations should therefore be directed in identifying sperm traits that affect the probability of fertilizing an egg under various circumstances.

Our findings so far suggest that sperm selection within a single ejaculate generally improves offspring performance across the life cycle. These results suggest that haploid selection can play a crucial role in zebrafish and possibly other species’ reproduction by for instance weeding out suboptimal male gametes, thereby ensuring the quality of the resulting offspring. Importantly, increased offspring fitness resulting from sperm selection within a male's ejaculate does not come at the cost of accelerated reproductive ageing. These findings have important implications for assisted reproductive techniques, such as in vitro fertilization and intracellular sperm injection that are widely used in the agricultural practice and the clinic because they essentially remove many aspects of within‐ejaculate sperm selection. In fact, a recent study in house mice *Mus musculus* found that selecting sperm through chemotaxis prior to intracellular sperm injection improved the outcome success rate (Pérez‐Cerezales et al. 2018). We therefore need more research into within‐ejaculate sperm selection across a broad variety of taxa because we are only beginning to understand the importance of this biological process for offspring fitness, ageing, and health.

Associate Editor: R. Snook

## Supporting information

Supporting Information.

## Data Availability

All related data will be made accessible in Dryad upon publication.
