# Supplementary material for: Selection for longer lived sperm within ejaculate reduces reproductive ageing in offspring
Source: Evol Lett. 2019 Feb 14;3(2):198–206. doi: 10.1002/evl3.101 (PMC6591544; doi:10.1002/evl3.101)
Supplement: Supplementary file 1 — Supporting Information. [file EVL3-3-198-s001.docx]

**Supplementary Material**

**For**

Selection for longer-lived sperm within ejaculate reduces

reproductive ageing in offspring

Ghazal Alavioon^1^, Andrea Cabrera Garcia^1^, Magali LeChatelier^1^, Alexei A. Maklakov^1,3^, Simone Immler^1,3^

^1^ Department of Ecology and Genetics, Evolutionary Biology Centre, Uppsala University, Norbyvägen 18D, 75 236 Uppsala, Sweden

^2^ School of Biological Sciences, University of East Anglia, Norwich Research Park, Norwich NR4 7TJ, United Kingdom

Material and methods

Animal model

For all experiments, we used zebrafish *Danio rerio* from the outbred wild-type AB strain originally purchased from ZIRC (Zebrafish International Resource Center, University of Oregon, Eugene,USA), kept and bred for up to two generations. following a strict outbreeding regime at the SciLifeLab zebrafish platform at Uppsala University (<http://www.scilifelab.se/facilities/zebrafish/>). The stuck fish (before entering the experiment) were kept in 3-liter or 9-liter tanks in a recirculating rack system (Aquatic Habitats, Z-Hab System) with 1:1 sex ratio at 26.4 ͦC ± 1.4 ͦC and a 12:12 diurnal light cycle. They were fed two to three times a day with a mixture of dry food (Aquatic Habitats, Zeigler adult zebrafish diet) and artemia (ZM Brineshrimp Cysts).

Experimental Set up

*In vitro fertilizations (IVF)*

All the fish used for IVF and outcrosses (at each aging point) were kept in 3-liter or 9-liter tank at densities of 15-20 and 45-50 fish respectively with mixed sex groups (on average 1:1 sex ratio). One day before IVF, the males were separated into unisexual groups of three and kept over night. The females were kept in breeding tanks with a company male using a separator (smell and visual contact). All tanks containing females were covered by a black cloth until next morning (to avoid light-induced-oviposition). Males and females were not fed for the twenty hours before the experiment to avoid faecal contamination of sperm and egg samples.

On the day of IVF females and males were anesthetized using 1.0-3.0 mg/L metomidate hydrochloride (Aquacalm^TM^) or in 0.16 g/L Tricaine methanesulfonate (Sigma-Aldrich MS222). Males were placed on a soft and wet sponge and squeezed gently in cranio-caudal direction to collect the ejaculate under dissecting microscope (Nikon SMZ800). From each male 0.7-0.8 μl of ejaculate was collected and transferred into a 0.2 ml Eppendorf tube containing 80 μl of Hank’s buffer (HBSS) and it was kept on ice for 5-10 minutes until IVF was performed. Females were placed on 15 cm Petri dishes and gently squeezed to obtain eggs. Clutches used for IVFs contained 20-300 high quality eggs and they were used within one minute after stripping.

*Sperm selection and IVFs*

To create the first generation (F1) we used split clutch design to perform in vitro fertilizations (IVFs). Sperm samples were very gently mixed and each ejaculate and egg clutch were divided into two parts. In each part the sperm were under one of two treatments (LAT or SAT).

In the long-activation time (LAT) treatment, 25 μl of ejaculate-Hank’s mix of a male were activated with 400 μl of water and added to one half of each clutch 25 seconds after activation to obtain a 50% decline in the amount of motile sperm (see Alavioon et al. 2017 for supporting data). In the short-activation treatment (SAT), 10 μl of ejaculate-Hank’s mix from the same male were mixed with extra 15 μl of Hanks (to compensate for osmolarity in Hanks to water ratio), activated with 400 μl water and added to the other sub clutch immediately after activation. Activated sperm in both treatments were added simultaneously to both sub clutches to avoid any egg effect. Eggs and sperm were mixed gently using a brush and eggs were transferred into a 15 cm Petri dish with Methylene blue solution (anti fungus) after 1 minute and 30 seconds. All IVFs were performed on a warming plate (Minitube HT50) at 28.5 °^C^ and all petri dishes were transformed to an incubator with 28.5 °^C^.

*Rearing F1 offspring*

The embryos resulting from IVFs were checked 2-4 hours post fertilization (hpf). Unfertilized and bad quality eggs were removed and about 70 embryos were transferred to a 9 cm Petri dish containing Methylene blue solution (anti fungus treatment). On day 5-6 post fertilization, 70 larvae from each family were randomly chosen and transferred into a 3-liter tank in the zebrafish system at the facility and reared until sexual maturity, then 10 males and 10 females (on average) were randomly selected and kept together until the time of the experiment. All fish were maintained until a maximum age of 24 months. Fish that survived until that age were then humanely euthanized with an overdose of MS222.

*Natural spawning*

Starting at the age of 12 months, experimental males and females from F1 were setup for natural spawnings with wildtype AB zebra fish to asses reproductive success. All the fish used for outcrosses with SAT and LAT F1 fish at each age point were wild type AB fish bred in the facility following a careful breeding regime to maintain outcrossing. They were maintained in 9-liter tanks at densities of 30-40 fish. The SAT and LAT fish were kept in 9-liter tanks at densities of 30-40 fish, both in mixed sex groups. At the age of 12 months, 2-4 males and 2-4 females were randomly selected from 26 LAT and 26 SAT families. The same families were chosen in each selection regime for the comparison of full siblings and reduce variation between families. Fish were individually tagged by using Visible Implant Elastomer (VIE) (BIOWEB) for tracking over time. Each fish was randomly assigned a wild type AB partner between 8-12 months of age for natural spawning. The same procedure was repeated on the same fish at age 15, 18 and 24 months, but every time we used a new partner fish between 8-12 months old. One day before natural spawning, males and females were randomly chosen from both selection and wild type lines. One male from either of the selection lines and one female from wild type lines or vice versa were kept in a breeding tank with a separator in between. The next morning the separator was taken out at 8:30-8:45 am to let the two fish spawn. Eggs were collected 2-3 hours after spawning. Both males and females were not fed for the last twenty hours before the experiment to avoid faecal contamination of embryos. In total, we were able to observe 116 fish from SAT and 111 fish from LAT at age 12 months; 105 fish from SAT and 107 fish from LAT at age 15 months; 98 fish from SAT and 99 fish from LAT at age 18 months; 77 fish from SAT and 76 fish from LAT at age 24 months. All tagged fish were individually followed and checked upon on a daily basis and natural or accidental death was recorded.

*Offspring traits measurements*

To calculate the fertilization success, eggs were checked within 1 hour post egg collection (~ 4 hours post fertilization). To calculate fertilization success and fecundity unfertilized and bad quality eggs were counted and removed. Survival rate was checked at 24 hours post fertilization. Seventy embryos were transferred to a 15 cm Petri dish containing Methylene blue solution and moved back to the incubator. All the traits were measured at 12, 15, 18 and 24 months.

*Statistical analysis*

Software R v. 3.3.0 (R Development Core Team. R, 2017). was used for all analysis. All binomial traits were analyzed assuming a binomial error distribution, whereas total egg production was analyzed assuming a Poisson error distribution. We used generalized linear mixed effect models for all analyses (glmer function in package *lme4* for the software R). The significance of the fitted model was assessed using analysis of variance (ANOVA) with type III sums of squares tested with an analysis of deviance on a chi-square distribution using package *car.*

We defined “family” and individual “ID” as a random factor and “Treatment”, “ALR” and “Age” as fixed factors. We analyzed data for males and females separately, because the traits that were measured represent different biological traits in the two sexes and are not directly comparable. All the interactions in the final models were assessed performing backward selection (removing model terms starting with interactions with highest order term). At each level, the model was compared to the previous model running Anova function in package *car*. Age and age of last reproduction (ALR) was scaled in all models and optimizer “bobyqa” was used in all models except total number of eggs (fecundity).

*Ethics permit*

All experiments described here are in accordance with the guidelines and approved by the Swedish Board of Agriculture (Jordbruksverket approval number C3/15).

Supplementary material reference list

R Development Core Team. R. 2017. R: A language and environment for statistical computing. Vienna, Austria: R Foundation for Statistical Computing.
